# Feedback-informed treatment in emergency psychiatry; a randomised controlled trial

**DOI:** 10.1186/s12888-016-0811-z

**Published:** 2016-04-19

**Authors:** Flip Jan van Oenen, Suzy Schipper, Rien Van, Robert Schoevers, Irene Visch, Jaap Peen, Jack Dekker

**Affiliations:** Arkin, Klaprozenweg 111, 1033 NN Amsterdam, The Netherlands; Universitair Medisch Centrum Groningen, Hanzeplein 1, 9713 GZ Groningen, The Netherlands; Klinische Psychologie Vrije Universiteit van Amsterdam, de Boelelaan 1105, 1081 HV Amsterdam, The Netherlands; Baarsjesweg 224, 1058 AA Amsterdam, The Netherlands

**Keywords:** Patient feedback, Randomised controlled trial, Crisis intervention, Efficacy, Outcome monitoring

## Abstract

**Background:**

Immediate patient feedback has been shown to improve outcomes for patients in mild distress but it is unclear whether psychiatric patients in severe distress benefit equally from feedback. This study investigates the efficacy of an immediate feedback instrument in the treatment of patients with acute and severe psychosocial or psychiatric problems referred in the middle of a crisis.

**Methods:**

A naturalistic mixed diagnosis sample of patients (*N* = 370) at a Psychiatric Emergency Centre was randomised to a Treatment-as-Usual (TAU) or a Feedback (FB) condition. In the FB condition, feedback on patient progress was provided on a session-by-session basis to both therapists and patients. Outcomes of the two treatment conditions were compared using repeated measures MANCOVA, Last Observation Carried Forward and multilevel analysis.

**Results:**

After 3 months, symptom improvement in FB (ES 0.60) did not significantly differ from TAU (ES 0.71) (*p* = 0.505). After 6 weeks, FB patients (ES 0.31) actually improved less than TAU patients (0.56) (*p* = 0.019).

**Conclusions:**

Patients with psychiatric problems and severe distress seeking emergency psychiatric help did not benefit from direct feedback.

**Trial registration:**

Dutch Trial Register, NTR3168, date of registration 1-9-2009

**Electronic supplementary material:**

The online version of this article (doi:10.1186/s12888-016-0811-z) contains supplementary material, which is available to authorized users.

## Background

Feedback systems have been developed in recent decades that provide therapists, patients or both with information about patient progress on a session-to-session basis [1–4]. The assumption in this ‘feedback-informed treatment’ is that clients feel more engaged in the therapy process and that therapists are better able to adapt their therapeutic approach when feedback information suggests that treatment is unsuccessful [3, 5, 6]. In a meta-analysis incorporating nine studies, Lambert & Shimokawa [7] found effect sizes varying from .23 to .33. They concluded that the number of psychotherapy patients who deteriorate in routine care (5–10 % in adult psychotherapy, 14–24 % in child psychotherapy) can be reduced by half using their feedback method.

Most feedback studies have been performed in psychotherapeutic settings and in samples of patients in mild distress who are generally not suffering from major psychiatric disorders.

The available studies in psychiatric samples in the last decade show that feedback improves outcomes for those with more severe mental health problems but that effect sizes are reduced [8]. However, feedback systems differ enormously with respect to the measures used and the frequency of administration [2], and the small number of studies and the heterogeneity of both the studies and the feedback systems make it hard to draw general conclusions [8].

Adding feedback may prove particularly valuable in this psychiatric population since the non-attendance levels in psychiatry are substantial, especially in the group with severe distress [5, 9]. Duncan et al. [5] state that patients who have lost their sense of mastery and their faith in therapy can be expected to feel empowered when their views and preferences are explicitly taken into account in a feedback process.

Since using feedback may prove to be a valuable tool in psychiatric treatment, we aimed to investigate, in an RCT, whether applying a feedback system can be effective in a psychiatric setting involving intensive outpatient care following a crisis evaluation.

## Method^1^

### Setting

The study setting was a Crisis Intervention & Brief Therapy team (CIBT team) in Amsterdam where patients with severe psychiatric and psychosocial problems are treated on an outpatient basis for a maximum of 6 months.

Patients are referred by GPs, mental-health workers and the police. Indication for treatment by the CIBT team is based upon the need for immediate help felt by the patient or referring professional. ‘Crisis’ is defined as: the patient needs help within 24 h due to a risk of suicide, serious behavioural problems, problems with the law and safety concerns, a sudden loss of social support and/or need for involuntary admission.

The CIBT works from a transdiagnostic perspective, which means that the assessment is not based solely on the diagnostic category but on the overall presentation of symptoms, and the needs and capacity of the patient and relatives. The need for acute help or treatment is integrated with a diagnostic screening and interventions are initiated immediately if necessary. The group of participating therapists consists of a highly experienced permanent staff of six psychiatrists, ten social psychiatric nurses, two psychologists and a family and marital therapist. In addition, the team includes a group of - on average - eight experienced and intensively supervised residents in psychiatry who each work at the CIBT for a period of six months. Clients are assigned to the duty therapist. No selection is made based on the diagnosis of the client or the discipline of the therapist. A total of 32 residents participated during the study period of about three years as a whole. The team uses a systemic approach that incorporates supportive and behavioural interventions [10]. All patients undergo a full clinical psychiatric examination. Treatment may involve pharmacotherapy and psycho-education and includes outreaching care if needed.

### Study design, randomisation and inclusion criteria

This study was designed as a randomised controlled trial in ‘routine emergency care’ comparing Treatment As Usual (TAU) with a Feedback condition (FB). The difference between TAU and FB is that, in every session in FB, feedback was obtained from the patients about progress in their functioning and about the therapeutic alliance [11], and this feedback was discussed by the therapist and the patient together. In the TAU condition, feedback was obtained every six weeks without feeding the results back to the patient or the therapist.

As the emergency setting made it impossible to distinguish in advance between patients who would be treated in the CIBT team and patients who would be referred to other treatment settings after the first contact, we conducted a pre-randomisation procedure for including patients in the study sample [12]. A random allocation sequence was generated using the SPSS random number generator. Patients were assigned to the FB or TAU condition by a research assistant who knew the allocation sequence but had no information about the patients.

### Intervention

Prior to the first session, a research assistant explained the principles of the feedback system, the Patient for Change Outcomes Management System (PCOMS), to all patients who had been randomised to the FB condition. Before each session, patients scored their well-being using the Outcome Rating Scale (ORS) and immediately received the printed score on a clipboard. The scores on the ORS form were discussed with the therapist at the beginning of each session. At the end of the session, the patient evaluated the therapy session using the Session Rating Scale (SRS) and also discussed the score with the therapist. When the crosses on the ‘What did you think of the session?’ form indicated reticence or plain dissatisfaction, the reasons for being dissatisfied were discussed with the therapist. When scores indicated general satisfaction, as indicated by a sum score exceeding 36 [13], the therapist asked for comments about how to improve the therapy.

The research assistant invited patients in the TAU condition to complete the ORS form at intake and every six weeks after that. The score was recorded in the database, and was not accessible to therapists or patients. In both conditions, patients were asked to complete the BSI and OQ45 questionnaires, first upon entering the service, and then every 6 weeks up to a maximum of 24 weeks.

### Training of therapists and application of feedback

Staff therapists were trained to administer, score and provide feedback to patients on the basis of the training manual provided for the ORS and SRS [14] before the study started. Follow-up supervision sessions were organised regularly during the course of the research project to maintain adherence. Therapists were trained to discuss the SRS score and encourage patients to express any comments and concerns about the session by making suggestions about how to improve collaboration and therefore address potential breaches in the alliance. Therapists were given the discretion to decide how to interpret and best integrate scores during the course of the treatment. However, if the ORS curve showed no improvement during the initial sessions, therapists were required to consult a colleague and consider other treatment options.

### Measures

#### Independent variables

The data collected at baseline (the emergency consultation) were: age, gender, domestic situation, ethnicity and main DSM IV diagnostic category.

#### Outcome measures

The number of therapy sessions and the duration of treatment were derived from the patient registration systems of Arkin Mental Health Care in Amsterdam. The link to the database of this system was established with an encrypted code based on gender, date of birth and the first two letters of the family name. This link made it possible to deduce data for unique patients.

#### Choice of feedback system

In meta-analyses, three elements which make feedback more effective were identified [2, 7]: when information about patient *progress* (by contrast with information about patient *status* only) was supplied, when feedback was reported frequently (more than twice over the course of treatment), and when both the patient and the therapist were informed about progress. A feedback system that incorporates these elements is the Partners for Change Outcome Management System (PCOMS) [13, 15]. An advantage of PCOMS is that it uses much shorter score lists than other systems, which is important for psychiatric patients with short attention spans. Three randomised controlled studies have been performed with PCOMS [16–18]. In these studies - which took place in student and family counselling settings - patients and couples in the feedback condition were found to improve more than patients receiving treatment as usual.

### The Partners for Change Outcome Management System (PCOMS)

PCOMS [13, 15] comprises two very short (VAS) scales consisting of four items each: firstly the Outcome Rating Scale (ORS) - which assesses change in three areas of client functioning: individual (or symptomatic) functioning, interpersonal relationships, and social role performance - and the Session Rating Scale (SRS) for scoring the quality of the working alliance.

The psychometric properties of the American and Dutch versions of this instrument have been evaluated [13, 15, 19, 20], resulting in coefficient alpha values ranging from 0.84 to 0.93 for the ORS and from 0.80 to 0.90 for the SRS for both the American and Dutch versions. ORS test-retest reliability coefficients (Pearson’s *r*) for both Dutch and American versions were reported ranging from 0.49 to 0.66, and from 0.49 to 0.65 for the SRS. With respect to this relatively weak test-retest reliability, Hafkenscheid et al. [19] point out that correlations between subsequent administrations are an inappropriate operational definition of test-retest reliability for instruments designed to be sensitive to a client’s perception of subjective change.

### Outcome Questionnaire 45 (OQ45)

The OQ45 [21] consists of 45 statements in three subscales that assess Symptom Distress (SD), Social-Role functioning (SR) and Interpersonal Relationships (IR). Jong et al. [22] conducted a psychometrical evaluation of the Dutch version of the questionnaire. Internal consistency (alpha) for the Total score obtained with the Dutch OQ-45 ranges from 0.92 to 0.96. Test-retest reliability (Pearsons’s r) ranges from 0.79 to 0.82.

### The Brief Symptom Inventory (BSI)

The BSI [23] is the concise version of the Symptom Checklist 90 (53 statements) for measuring symptoms of psychopathology in adults. Reliability (alpha coefficient) for the Dutch version of the scale as a whole is .96 [24]; test-retest reliability (Pearons’s r) is 0.90.

### Attitude survey

In order to check for bias resulting from changes in therapists’ attitudes to applying feedback, therapists were asked to complete an attitude survey [16] at the start and finish of the study consisting of 19 statements reflecting therapist opinions about PCOMS, examples being ‘I consider this instrument useful’ or ‘I don’t think this instrument is useful for clients’. This survey has not been evaluated psychometrically (Additional file 1).

Changes in attitudes towards the feedback process were tested between baseline and 12 weeks (paired *t*-test).

### Adherence survey

After one year (halfway through the study,) staff therapists were asked, in order to check for bias in the results due to lack of adherence, to complete an anonymous survey about the extent to which they had been able to apply the feedback as intended (Additional file 2).

This survey was designed by the first author and contains two items:

- the percentage of sessions in which the therapists applied the feedback measures adequately (results categorised in: ‘10–40 %’, ‘40–70 % and ‘more than 70 % of the sessions adequately applied’);
- the time spent (in minutes) discussing the ORS;
- the time spent discussing the SRS.

### Data analysis

#### Sample size calculation

With two groups of 90 patients, an alpha of 0.05 (one-tailed), an effect size of about 0.3 on the BSI total score (Global Severity Index) at 12 weeks (mean EXP group = 1.0; mean TAU = 1.3; standard deviation at week 12 is 0.80) can be detected with a statistical power of 80 %. Analysis was performed using to the intention-to-treat principle. Sample size was calculated a priori. No separate power analysis was performed for the ORS and OQ45 since the BSI was the primary outcome measure, as established beforehand [11].

Baseline characteristics were compared using Chi-square tests, ANOVA and Mann-Whitney tests. The proportions of early treatment termination and non-response (patients still in treatment without measurement) were compared at 6, 12, 18 and 24 weeks using Chi-square tests. To test for selective drop-out at these measurement points, patient characteristics (including diagnostic categories), baseline measurements and the number of sessions were compared.

Outcomes of the two treatment conditions (observed cases) were compared using repeated measures MANCOVA with the number of sessions as a covariate. In this analysis, each subsequent measurement was compared separately with the baseline measurement. An identical analysis was performed on a dataset on which Last Observation Carried Forward (LOCF) had been performed.

Furthermore, multilevel analysis (MLwiN v2.25) was used to establish time by treatment interactions in ORS, BSI and OQ45. Three levels were included: patient, therapist and time. First, the measurements from start to week 12 (T0,T6,T12) were analysed, followed by the measurements from start to week 24 (T0,T6,T12,T18,T24). The number of sessions was included as a covariate.

Analyses conducted to compare the outcomes of completed treatments were based primarily on observed cases. LOCF and multilevel analysis were used as an additional method, primarily with the aim of comparing the results of terminated treatments with different durations. Secondly, both LOCF and multilevel analysis were used to handle data that were incomplete due to missing scores and ‘drop-out’ (in other words, clients who terminated treatment without mutual consent).

Pretreatment-posttreatment effect sizes for each treatment group were calculated by dividing the mean difference by the pooled standard deviation of the baseline measurement and the measurement point concerned.

In addition, the numbers of patients profiting from treatment in both conditions were compared. Based on Cohen’s d [25] a cut-off was established at an effect size of 0.5 (which means a ‘medium’ effect). Clients showing an increase > 0.5 SD on GSI were classified as ‘improved’, clients showing an increase < 0.5 SD as ‘not changed’ and clients dropping > 0.5 SD as ‘deteriorated’. In all analyses α = 0.05 (two-sided) was used as the level of significance. All statistical analyses, except multilevel analysis, were conducted in SPSS 17.0.

## Results^2^

### Patient sample

**Fig. 1:**
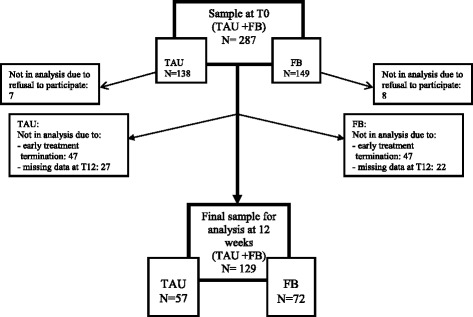
Participant flow

Between 2009 and 2012, a total of 861 patients were referred to the Psychiatric Emergency Centre. The 222 patients who were unable to fill out a questionnaire at intake were excluded. A group of 269 patients were offered only one session for crisis evaluation, resulting in either immediate admission to a psychiatric hospital or referral to the patient's own general practitioner/therapist (when no indication for acute psychiatric help was found). In 370 patients the crisis intervention was followed by brief therapy, which was defined as more than two sessions (including the first crisis evaluation session). Of these patients, 83 terminated treatment within six weeks, making it impossible to assess their progress at the first time point (T6). The study sample therefore included 287 patients (Fig. 1). As 94 patients terminated treatment before T12, 49 (17.1 %) did not complete the questionnaires at this time and 15 (5.2 %) refused to participate, a total of 129 patients had received either TAU (57) or TAU + FB (72) at 12 weeks.

In conclusion, score evaluation at T6 and T12 was not possible for some of the study sample of 287 patients. LOCF and multilevel analyses were performed to correct for missing data.

### Sample characteristics and representativeness testing

Of the participants in the total study sample (*n* = 287) – FB and TAU conditions combined – 135 (47 %) were men and 152 (52 %) were women. The mean age was 38 years, with the majority (58 %) being in the 30–49 age category (Table 1). The most common diagnostic categories were adjustment disorder (21 %), depression (19 %) and psychosis (15 %). About 40 % (42 %) of the patients were Dutch-born; about 60 % had their roots elsewhere. A substantial proportion (45 %) were living alone. On average, patients suffered from severe distress, as indicated by a mean BSI score of 1.84 at T0, which is significantly lower than the mean BSI score in Dutch clinical populations (1.23) found by de Beurs [24]. No differences for any baseline characteristic (including diagnostic categories) were found (Table 1), indicating a successful randomisation procedure.

**Table 1:** Baseline characteristics of TAU versus FB condition at T0 (measurement at start of treatment)

Variable	TAU condition (*n* = 138)	FB condition (*n* = 149)	Total group (*n* = 287)	p*
Age, mean (sd)	38.1 (11.31)	38.2 (11.20)	38.1 (11.24)	0.934
Age subgroups				0.970
Gender, n (%):				0.346
-male	69 (50.0)	66 (44.3)	135 (47.0)	
-female	69 (50.0)	83 (55.7)	152 (53.0)	
Cultural background, n (%):				0.522
- Dutch	54 (39.1)	67 (45.0)	121 (42.2)	
- Surinam	7 (5.1)	8 (5.4)	15 (5.2)	
- Turkish	2 (1.4)	5 (3.4)	7 (2.4)	
- Moroccan	11 (8.0)	6 (4.0)	17 (5.9)	
- other	28 (20.3)	31 (20.8)	59 (20.6)	
- unknown	36 (26.1)	32 (21.5)	68 (23.7)	
Living situation, n (%):				0.181
- alone	56 (40.6)	74 (49.7)	130 (45.3)	
- with children without partner	9 (6.5)	13 (8.7)	22 (7.7)	
- with parents in family	10 (7.2)	3 (2.0)	13 (4.5)	
- with partner	32 (23.2)	25 (16.8)	57 (19.9)	
- other	14 (10.1)	15 (10.1)	59 (20.6)	
- unknown	17 (12.3)	19 (12.8)	36 (12.5)	
Well-being/Severity of complaints at T0. mean (SD):				
GSI	1.87 (0.84)	1.80 (0.90)	1.84 (0.87)	0.453
OQ 45 total score	93.99 (27.46)	90.18 (29.31)	91.97 (28.46)	0.302
ORS	12.84 (8.76)	13.36 (9.21)	13.10 (8.99)	0.628
Diagnosis:				0.663
- psychotic disorder	23 (16.7)	21 (14.1)	44 (15.3)	
- depression	29 (21.0)	25 (16.8)	54 (18.8)	
- adjustment disorder	29 (21.0)	31 (20.8)	60 (20.9)	
- personality disorder	15 (10.9)	13 (8.7)	28 (9.8)	
- psychosocial problems	8 (5.8)	8 (5.4)	16 (5.6)	
- other	30 (21.7)	42 (28.2)	72 (25.1)	
- unknown	4 (2.9)	9 (6.0)	13 (4.5)	
Referring service:				0.626
GP	14 (10.1)	21 (14.1)	35 (12.2)	
Mental health Service	18 (13.0)	22 (14.8)	40 (13.9)	
Patient	4 (2.9)	9 (6.0)	13 (4.5)	
Family/friends	70 (50.7)	63 (42.3)	133 (46.3)	
ER	2 (1.4)	3 (2.0)	5 (1.7)	
Public Health (GGD)	2 (1.4)	3 (2.0)	5 (1.7)	
Other	19 (13.8)	15 (10.1)	34 (11.8)	
Unknown	9 (6.5)	13 (8.7)	22 (7.7)	

* ‘Unknown’ is excluded in p-analysis

The average number of treatment sessions offered to all patients was 9.3 (SD 5.05). No differences were found between conditions. The average duration of treatment was 105 days (range 0–231 days). The majority of patients (49.9 %) ended treatment within three months, two-thirds of patients (55.8 %) finished treatment within eight sessions and half of all patients (49.5 %) had 4–8 sessions. There were no significant differences in treatment duration between the two conditions: the mean was 105.4 days (SD 51.81) for patients in TAU and 103.5 (SD 50.23) days for patients in FB. In addition, no relationship was found between diagnostic categories and the average number of sessions or duration of treatment (data not shown).

### Does systematic session-by-session feedback improve outcome?

After six weeks of treatment, patients in the TAU condition – based on observed cases analysis – had improved by .58 on the GSI score (from 1.88 at T0 to 1.30 at T6); patient scores in the FB condition had improved by .31 (from 1.80 at T0 to 1.51 at T6). Patients in TAU achieved significantly higher treatment gains than patients in FB (*p* = 0.020). LOCF (*p* = 0.021) and multilevel analysis (*p* = 0.006) produced similar GSI results at six weeks (Table 2).

**Table 2:** The efficacy of the feedback interventions Total scores for BSI (GSI score), Q 45 and ORS, observed cases (OC), Last Observation Carried Forward (LOCF) and Multilevel analyses (ML)

Time in weeks	TAU Mean score (sd), N	FB Mean score (sd), N	p OC	p (F) LOCF	p ML
	GSI				
0 wks	1.88 (0.84) *N* = 138	1.80 (0.90) *N* = 149	ns	ns	ns
6 wks*	1.30 (0.82) *N* = 92	1.51 (0.90) *N* = 96	**0.020**	**0.021**	**0.006**
12 wks**	1.26 (0.81) *N* = 57	1.22 (0.84) *N* = 72	ns	ns	ns
18 wks	1.35 (0.85) *N* = 35	1.25 (0.79) *N* = 36	ns	ns	ns
24 wks	0.92 (0.77) *N* = 22	1.07 (0.66) *N* = 30	ns	ns	ns
	OQ45 (total score)				
0 wks	93.99 (27.46) *N* = 113	90.18 (29.31) *N* = 127	ns	ns	ns
6 wks	82.35 (28.68) *N* = 71	85.61 (29.42) *N* = 71	0.065	**0.035 (4.476)**	**0.047**
12 wks	81.98 (25.59) *N* = 44	79.56 (29.13) *N* = 59	ns	ns	ns
18 wks	77.82 (33.03) *N* = 25	77.55 (29.72) *N* = 21	ns	ns	ns
24 wks	71.47 (30.73) *N* = 16	67.49 (27.14) *N* = 20	ns	ns	ns
	ORS				
0 wks	12.8 (8.76) *N* = 134	13.4 (9.21) *N* = 148	ns	ns	ns
6 wks	18.7 (8.94) *N* = 86	18.1 (10.83) *N* = 130	ns	ns	ns
12 wks	19.8 (8.88) *N* = 57	17.8 (9.54) *N* = 91	ns	ns	*0.0503*
18 wks	19.0 (9.81) *N* = 30	19.3 (9.73) *N* = 55	ns	ns	ns
24 wks	20.0 (8.31) *N* = 21	21.7 (10.48) *N* = 37	ns	ns	ns

Mean scores refer to Observed Cases

Multilevel analyses, taking into account the levels of the patient, therapist and time, showing time by treatment interactions in ORS, BSI and OQ45 (from start to T6, T12,T18,T24)

Data in bold are significant scores; score favouring FB is indicated in italics

*99 clients (34.5 %) did not fill in forms at T6 despite being in treatment

**64 clients (22.3 %) did not fill in forms at T12 despite being in treatment

The OQ45 total score at 6 weeks was significantly different with LOCF (*p* = 0.035) and multilevel analysis (*p* = 0.047), and also favoured TAU.

At the predetermined primary measurement point – the GSI at 12 weeks [11] – no significant difference in treatment gains was found between the conditions: on the basis of observed cases, patients in the TAU condition reported mean treatment gains of 0.62 (GSI decreased from 1.88 at T0 to 1.26 at T12); this gain was 0.58 in the FB condition (GSI decreased from 1.80 at T0 to 1.22 at T12). The ORS score at 12 weeks did not indicate any significant difference favouring FB. LOCF and multilevel analysis also produced no significant differences or trends in total scores at 12 weeks.

Table 3 shows that patients in TAU at 6 weeks did significantly better on the BSI subscale (depression, hostility, somatic complaints and anxiety) and the OQ 45 subscale (severity). Six other scales/subscales did indicate not-significant differences favouring TAU (from *p* = 0.053 to *p* = 0.093). There were only two subscales that did not significantly favour FB (*p* = 0.055 and *p* = 0.069).

**Table 3:** The efficacy of the feedback interventions Significant outcomes and trends in BSI subscales and OQ45 subscales based on observed cases, LOCF analyses and Multilevel (ML) analyses

Weeks	Subscale	TAU Mean score (sd), N	FB Mean score (sd), N	p OC	p (F) LOCF	p ML
	BSI					
6 wks	Depression	1.70 (1.11) *N* = 91	2.04 (1.21) *N* = 96	**0.043**	**0.037 (4.371)**	**0.008**
	Hostility	0.91 (1.04) *N* = 93	1.09 (0.99) *N* = 96	**0.018**	**0.018 (5.676)**	**0.013**
	Somatic	0.94 (0.81) *N* = 91	1.21 (0.96) *N* = 96	0.079	0.079	**0.026**
	Cognitive	1.66 (1.04) *N* = 92	1.87 (1.09) *N* = 96	0.088	0.087	0.065
	Anxiety	1.45 (1.00) *N* = 92	1.70 (1.17) *N* = 96	0.060	0.067	**0.028**
	Interpersonal	1.36 (1.08) *N* = 92	1.47 (1.11) *N* = 96	ns	ns	0.084
12 wks	Interpersonal	1.22 (0.92) *N* = 58	1.28 (1.00) *N* = 72	0.071	ns	0.075
18 wks	Hostility	1.07 (1.11) *N* = 35	0.77 (0.86) *N* = 36	*0.069*	ns	Ns
24 wks	Depression	1.11 (0.90) *N* = 21	1.56 (0.95) *N* = 30	ns	0.055	0.070
	OQ45					
6 wks	Severity	44.92 (17.01) *N* = 71	47.17 (18.03) *N* = 72	**0.035**	**0.017 (5.780)**	**0.024**
12 wks	Socially	15.63 (4.51) *N* = 37	16.28 (6.18) *N* = 52	0.083	ns	0.093
	Severity	44.03 (15.75) *N* = 44	43.80 (16.88) *N* = 60	ns	*0.055*	Ns
24 wks	Severity	36.57 (17.90) *N* = 16	38.10 (16.46) *N* = 20	ns	0.053	Ns

Mean scores refer to observed cases

All subscores favour the TAU condition, with the exception of the BSI Hostility score (only OC) at 18 weeks and O 45 severity at 12 weeks (only LOCF); scores favouring FB are indicated in italics, data in bold are significant scores

Multilevel analyses, taking into account the levels of the patient, therapist and time, showed time by treatment interactions in ORS, BSI and OQ45 (from start to T6, T12,T18,T24)

**Table 4:** Deterioration, no change and improvement based on GSI

		TAU		FB		Total		Comparison TAU and FB:	
		N	%	N	%	N	%	Chi2	*p*
T6	deterioration	5	5.4	9	9.4	14	7.4	7.645	**0.007**
	no change	40	43.5	56	59.4	96	51.1		
	Improved	47	51.1	31	32.3	78	41.5		
T12	deterioration	3	5.3	6	8.30	9,0	7.0	.115	0.360
	no change	22	38.6	29	40.3	51	39.5		
	Improved	32	56.1	37	51.4	69	53.5		
T18	deterioration	1	2.9	3	8.3	4	5.6	.688	.205
	no change	17	48.6	11	30.6	28	39.4		
	Improved	17	48.6	22	61.1	39	54.9		
T24	deterioration	1	4.5			1	1.9	.234	.562
	no change	6	27.3	9	30.0	15	28.8		
	Improved	15	68.2	21	70.0	36	69.2		

Improved: increase > 0.5 SD on GSI

No change: improvement < 0.5 SD on GSI

Deterioration: dropping > 0.5 SD on GSI

Comparison TAU versus FB:% improved (> .5 SD increase) and % not improved (< .5 SD increase, i.e. ‘no change’ and ‘deterioration’ combined) in both conditions were compared. Data in bold are significant scores

To test for selective treatment termination in the first period, we looked for differences between the total study sample at 12 weeks (*N* = 129) and the group of clients eliminated from analysis due to early treatment termination or not filling out the forms (*N* = 158). This check involved the same items, plus the number of sessions and duration of treatment. Furthermore, we looked for differences between TAU (*N* = 57) and FB (*N* = 72) at 12 weeks, checking for the percentage of non-responding clients still in treatment (*N* = 64) as well as the percentage of all non-responding clients, including those who terminated treatment before 12 weeks (*N* = 158). We also checked for differences between the two conditions in terms of the percentage of clients who terminated the treatment without mutual consent (in other words, drop-out patients), looking at the total percentages in both conditions and at the separate measurement points. Neither of these comparisons revealed significant differences, suggesting that differences in treatment gains between TAU and FB were not affected by selective early treatment termination, missing data or drop-out.

### What do the differences in treatment gains mean in clinical practice?

To identify the significance of the differences in treatment gains for clinical practice, final outcomes were categorised according to the percentages of patients who did and did not benefit from treatment according to the GSI (Table 4).

At T6, significantly (*p* = 0.006) more patients in FB (68.8 %) had undergone no change or deterioration (in other words, their scores fell by > .5 SD on GSI) than in TAU (48.9 %). At twelve weeks these differences were no longer statistically significant: in FB 48.6 % of the patients showed ‘no change’ or ‘deterioration’, as opposed to 45.7 % in TAU. In the full sample 52.7 % of patients had improved at T12 and 67.3 % had done so at T24.

Table 5 presents the effect sizes (ES) of treatment.

**Table 5:** Effect sizes TAU and FB on different measuring points, based on GSI scores

(d, N, sd)	TAU	EXP	Full sample	F	p
ES GSI T6	0.56 (*N* = 92; sd0.70)	0.31 (*N* = 96; sd0.77)	0.44 (*N* = 188; sd0.74)	5.575	**0.019**
ES GSI T12	0.73 (*N* = 57; sd0.88)	0.62 (*N* = 72; sd0.95)	0.67 (*N* = 129; sd0.92)	0.446	0.505
ES GSI T18	0.60 (*N* = 35; sd0.77)	0.70 (*N* = 36; sd0.74)	0.65 (*N* = 71; sd0.75)	0.275	0.602
ES GSI T24	1.13 (*N* = 22; sd1.20)	0.86 (*N* = 30; sd0.82)	0.98 (*N* = 52; sd0.99)	0.921	0.342

$$ \mathrm{d}=\frac{\mathrm{Estimate}\kern0.5em \mathrm{T}\mathrm{x}\hbox{-} \mathrm{Estimate}\kern0.5em \mathrm{pretreatment}}{\left\{{\left(\mathrm{S}\mathrm{D}\kern0.5em \mathrm{pretreatment}\right)}^2+{\left(\mathrm{S}\mathrm{D}\kern0.5em \mathrm{T}\mathrm{x}\right)}^2\right\}/2} $$

Data in bold are significant scores

The ES for the TAU group at week 6 was .56 (sd 0.70), as opposed to .31 (sd 0.76) for the FB group, which is a significant difference in favour of the TAU group (*p* = 0.019). There were no significant differences in ES at other measuring points.

### Therapist attitude and adherence

Fifty-one therapists (19 staff members and 32 residents) completed an adherence and attitude survey at both the beginning and the end of the study. The mean score at the beginning was 73.88 (SD 9.29) out of 95 and 71.96 (SD 8.01) at the end, indicating that therapists’ attitudes to feedback were very positive on average, even though the initially high motivation of the therapists eroded slightly, albeit not significantly, over time (*p* = 0.06). In the adherence survey, 67 % of the staff therapists reported that they had applied PCOMS adequately in more than 70 % of the sessions; 14 % had applied it in 40–70 % of the sessions, and 19 % in 10–40 %. On average, therapists (*N* = 21) estimated that they spent 3.5 min on the ORS and 4 min on the SRS. Almost all patients completed the ORS and SRS forms: only one patient did not fill out a single ORS form, and two patients did not fill out a single SRS form.

## Discussion

This study was set up to determine whether the positive results of immediate feedback described in psychotherapy studies could also be demonstrated in short-term psychiatric treatment delivered in an outpatient emergency centre for a range of problems and disorders. Contrary to what we expected, we found no positive effect of immediate feedback at the predetermined end point of our study at twelve weeks. Furthermore, the effect was negative at six weeks because there was significantly less improvement in the FB condition than in the TAU condition. More patients in the FB condition were ‘not on track’ (showing no change or deterioration) during the treatment process. No difference was found between the TAU and FB groups with respect to duration and number of sessions. Comparing non-responding clients revealed no significant differences, suggesting that providing feedback did not influence selective drop-out in a positive way.

It can be concluded that we found no advantage to including feedback in an emergency psychiatry setting. This result clearly contrasts with most of the earlier studies of PCOMS, which have found substantial benefits with feedback in other treatment settings [16–18]. Three possible clinical explanations can be offered for our findings.

### Reduced ability to reflect during crisis

Characteristically, in crisis situations, people’s ability to consider alternatives and reflect on their situation is impaired [10]. Since the ability to reflect and consider alternatives are precisely the elements needed to benefit from feedback, it is plausible that the impairment of those abilities is responsible (in whole or in part) for the lack of effect of feedback.

Apart from this, patients in crisis desperately look for solutions, including someone who can offer a way out. Explicitly stressing shared responsibilities, as well as possibly introducing insecurity about different treatment options and outcome at the outset in a crisis situation might actually burden the therapeutic relationship. Formalising feedback may disturb the process of subtly balancing between sharing responsibilities for the content of treatment and taking responsibility for the form of the treatment process, which is part of the art of crisis intervention [10].

The finding that differences in favour of TAU emerged in the first six weeks can be interpreted in line with this explanation since, in the initial weeks, a crisis is more severe and the ability to reflect is poor. Later on – as patients stabilise more – immediate feedback is probably more acceptable to patients, even though it still does not lead to better outcomes.

### Low level of functioning and severity of psychiatric problems interfere with feedback effects

Simon et al. [26] found that feedback had less effect (d = .12 versus d = .30) in a sample with lower pre-test scores (OQ score 83.72) than in a sample with higher pre-test scores (mean OQ 88.8) in a previous study in the same clinic [27]. They suggested that the difference in pre-test scores could account for the reduced effect of feedback, and that ‘feedback interventions do not work as well with more disturbed patients as with the less disturbed’.

The pre-test scores found in our study indicate a higher level of distress than the pre-test scores in other feedback studies. The average ORS score was 13.3 (sd 9.14), as compared with 18.33 to 23.7 in other studies [15–18]; the mean pre-test OQ45 sum score was 91.78 (sd 27.80), as compared with 68 to 78 in other studies [28–30] (a lower OQ score means less severe complaints).

The low pre-test scores in our study may have been confronting for patients in FB and discouraging for patients who dysfunction in several life domains. Any positive effect of the feedback process would not seem to compensate for this drawback.

### Relatively high efficacy of TAU

It should be noted that the effect size in the TAU condition in our study (0.71 at twelve weeks) is relatively high by comparison with the TAU groups in other feedback studies: Harmon et al. [29] report 0.43, Hawkins [27] .63 and Reese [18] .38. It is possible that this did not leave an adequate margin for further improvement as a result of adding feedback to this treatment.

### Method: strengths and limitations

#### Limitations

This study took place in a naturalistic crisis setting and the implementation of the study was therefore challenging in several ways. Firstly, a pre-randomisation procedure had to be conducted instead of random assignment with a full evaluation of inclusion and exclusion criteria before initiating randomisation. Secondly, therapies inevitably differed in duration and intensity, and there was sometimes a change of therapist in the course of therapy. However, analyses based on observed cases, LOCF and multilevel analysis – with the latter two adjusting for missing data – lead to consistent results, suggesting that the overall conclusions are sound. A drawback in the design was that patients in the TAU group completed the ORS forms only every six weeks (to prevent bias coming from frequent ‘feedback-alike’ reflection on progress in TAU group), making it impossible to compare on-track/not-on-track trajectories in the two conditions. As a consequence, no conclusions can be drawn about the specific effect of feedback on the group of not-on-track patients. Nevertheless, the finding that a comparison of early termination and non-response in both conditions did not reveal differences suggests that early identification of not-on-track patients did not improve outcomes.

Another limitation is that data was not collected about co-existing treatment and the use of medication during the study and so it is not known whether these factors have influenced the outcome or selective drop-out. Even so, no differences were found in the drop-out rates for the different diagnostic categories and it therefore seems unlikely that medication use – which is generally linked to diagnostic categories – affected the outcomes. Sub-analyses by diagnostic group did not reveal significant differences. However, observed power was limited and so it is difficult to draw conclusions from these analyses. Finally, the adherence of the therapist to the feedback model was monitored by self-report and peer supervision but not measured systematically.

#### Strengths

The setting and population of this study are unique. No feedback study has yet been performed to our knowledge of patients in crisis suffering from severe psychiatric and psychosocial problems. The design ensures that possible differences in therapist characteristics are not responsible for differences in outcome: patients were allocated randomly to the different study conditions, and therapists – all of whom were experienced and qualified care providers – participated in both conditions and therefore treated approximately 50 % of their patients using PCOMS and 50 % on the basis of TAU. Given the fact that differences between therapists are usually more pronounced than differences between therapeutic methods, it is important to eliminate the therapist variable [31]. The flip side to this strength is a possible spill-over effect that may occur if therapists fail to distinguish clearly between both conditions. Another benefit of this design is that it is not very likely that allegiance factors (in other words, therapists believing in the effect of feedback) affected outcomes because therapists with both higher and lower levels of motivation delivered treatment to the Feedback condition.

A final strength of the study is that, contrary to previous studies, independent outcome measures (BSI and Q45) were provided instead of using the feedback measure (ORS) itself as an outcome measure. Providing outcome information to patients may result in ‘demand characteristics’ (patients responding to incidental hints about the therapists’ expectations) that favour the feedback condition [32, 33]. In line with this, Janse [34] has recently argued that, although PCOMS is a useful feedback instrument, its validity is limited and therefore other instruments should be added to corroborate progress. Ideally, studies should therefore use an independent outcome measure that is not discussed with the therapist.

In our study, the adverse effect of applying feedback would not have been revealed if BSI and OQ45 had not been added. This finding could suggest that ORS outcomes have indeed been influenced by ‘socially desirable’ scoring.

## Conclusions

To our knowledge, this is the first study suggesting that immediate progress feedback in psychiatric practice does not improve outcome and that it may even be counterproductive.

Perhaps patients did not benefit from feedback because they were unable or reluctant to think about the treatment process, and confronting them repeatedly with their low level of functioning may have demoralised them. If this is true, it may be better not to subject some patients with immediate feedback. Future research could determine whether pre-treatment functioning and the patient’s ability to reflect influence the success of feedback. In studies of this kind, independent outcome measures should be added to control for ‘socially desirable’ scoring during the feedback process.

### Availability of data and materials

The data and materials used in this study are available on request.

### Consent for publication

Not applicable.

### Ethics approval and consent to participate

The study protocol and informed consent procedure were evaluated in 2009 by the ethics committee for Dutch Mental Health Institutions, (Kamer Noord of the METiGG) (approval nr. 9219, 1-9-2009). Following their conclusion, the Committee concluded that, since feedback does not fall under the jurisdiction of the WMO (the Dutch law on scientific medical research on human subjects), the regular clinical procedure for informed consent at the department could be followed. The study was then explained to the patients, written information was provided and patients were asked to participate on a voluntary basis, which was noted in the medical file.

## Additional files

Additional file 1: Attitude survey. Therapist’s attitude to applying feedback (DOCX 18 kb) Additional file 2: Adherence Survey. Adherence of therapists to feedback method (DOCX 14 kb)

## Abbreviations

BSI: Brief Symptom Inventory
CIBT: Crisis Intervention & Brief Therapy team
FB: feedback condition
GSI: Global Severity Index
LOCF: Last Observation Carried Forward
OQ45: Outcome Questionnaire 45
ORS: Outcome Rating Scale
PCOMS: Patient for Change Outcomes Management System
SRS: Session Rating Scale
TAU: treatment-as-usual condition

## Footnotes

1 For a more detailed description of treatment elements, randomisation procedure, training of therapists and interpretation of feedback measures, see van Oenen et al. [10, 11].

2 Scores for alliance scales (Session Rating Scale and HAQ2) will be reported and discussed in a separate paper.
