# Supplementary material for: Feedback-informed treatment in emergency psychiatry; a randomised controlled trial
Source: BMC Psychiatry. 2016 Apr 19;16:110. doi: 10.1186/s12888-016-0811-z (PMC4837581; doi:10.1186/s12888-016-0811-z)
Supplement: Additional file 1: — Attitude survey. Therapist’s attitude to applying feedback (DOCX 18 kb) [file 12888_2016_811_MOESM1_ESM.docx]

**Attitude Survey ten aanzien van clientenfeedback**

**‘Wat is jouw gevoel over de Beterwetermeter?’**

Gebaseerd op:

vragenlijst attitude cliënt feedback Morten Anker, psycholoog

Vertaling: E. van Not (juli 2009)

**Naam: ……………….. Werkteam: .................... Datum : ….-….-….**

Er zijn aanwijzingen dat het verzamelen en bespreken van feedback van de client over de behandeling een positief effect heeft op de behandelresultaten. In de Beterwetermeter worden twee formulieren gebruikt om feedback te verkrijgen: het ORS formulier (“Hoe gaat het met u?”) voor het begin en de SRS (“Wat vond u van de bijeenkomst?”) voor het einde van de sessie. De ORS geeft de indruk van de cliënt weer over de samenwerking met de behandelaar; de ORS geeft de veranderingen in het welbevinden van de cliënt in de loop van de behandeling weer. De scores van de ORS worden, zoals je weet, gebruikt om veranderingen of ontbrekende verandering tussen de sessies te signaleren.

We zijn benieuwd naar je mening over het gebruik van deze formulieren in je behandelingen.

We vragen je jouw algemene indruk weer te geven, zoals je die op dit moment hebt; deze indruk kan zowel gebaseerd zijn op praktische ervaringen als op verwachtingen als op (principiële) opvattingen die je over het gebruik van feedback-instrumenten zoals de Beterwetermeter hebt.

1. Ik denk dat deze formulieren nuttig kunnen zijn voor mijn behandelingen…

| □ | □ | □ | □ | □ |
| --- | --- | --- | --- | --- |
| Volledig mee eens | Grotendeels mee eens | Weet niet | Grotendeels niet mee eens | Volstrekt niet mee eens |

2. Ik denk niet dat het voor cliënten nuttig is als therapeuten zich door dergelijke feedback laten leiden…

| □ | □ | □ | □ | □ |
| --- | --- | --- | --- | --- |
| Volledig mee eens | Grotendeels mee eens | Weet niet | Grotendeels niet mee eens | Volstrekt niet mee eens |

3. De doelstelling en de praktische uitwerking van de BWM spreken mij erg aan zodat ik sterk geneigd ben deze formulieren bij mijn behandelingen te gebruiken

| □ | □ | □ | □ | □ |
| --- | --- | --- | --- | --- |
| Volledig mee eens | Grotendeels mee eens | Weet niet | Grotendeels niet mee eens | Volstrekt niet mee eens |

4. Ik denk dat dit soort formulieren eerder storend dan nuttig bij de therapie zijn.

| □ | □ | □ | □ | □ |
| --- | --- | --- | --- | --- |
| Volledig mee eens | Grotendeels mee eens | Weet niet | Grotendeels niet mee eens | Volstrekt niet mee eens |

5. Ik denk dat ik dit soort feedback beter door middel van een gesprek met mijn cliënten kan verkrijgen.

| □ | □ | □ | □ | □ |
| --- | --- | --- | --- | --- |
| Volledig mee eens | Grotendeels mee eens | Weet niet | Grotendeels niet mee eens | Volstrekt niet mee eens |

6. Ik vind het belangrijk om met behulp van gevalideerde (d.w.z. bewezen deugdelijke en betrouwbare) formulieren mijn behandelkwaliteit te verbeteren.

,

| □ | □ | □ | □ | □ |
| --- | --- | --- | --- | --- |
| Volledig mee eens | Grotendeels mee eens | Weet niet | Grotendeels niet mee eens | Volstrekt niet mee eens |

7. Vanwege mijn opleiding,ervaring en/of begeleiding heb ik al voldoende kennis in huis over hoe ik moet behandelen. Ik heb daarom geen behoefte aan dit soort formulieren.

| □ | □ | □ | □ | □ |
| --- | --- | --- | --- | --- |
| Volledig mee eens | Grotendeels mee eens | Weet niet | Grotendeels niet mee eens | Volstrekt niet mee eens |

8. Ik kan, zonder dit soort formulieren te gebruiken, snel genoeg aanvoelen of cliënten zich begrepen voelen en of ze het idee hebben dat de gekozen werkwijze bij hen past.

| □ | □ | □ | □ | □ |
| --- | --- | --- | --- | --- |
| Volledig mee eens | Grotendeels mee eens | Weet niet | Grotendeels niet mee eens | Volstrekt niet mee eens |

9. Ik denk dat deze formulieren mij kunnen helpen om vast te stellen of de behandeling mijn cliënten helpt.

| □ | □ | □ | □ | □ |
| --- | --- | --- | --- | --- |
| Volledig mee eens | Grotendeels mee eens | Weet niet | Grotendeels niet mee eens | Volstrekt niet mee eens |

10. Ik denk dat veel van mijn cliënten zullen weigeren om deze formulieren in te vullen.

| □ | □ | □ | □ | □ |
| --- | --- | --- | --- | --- |
| Volledig mee eens | Grotendeels mee eens | Weet niet | Grotendeels niet mee eens | Volstrekt niet mee eens |

11. Ik denk dat deze formulieren alleen in sommige, speciale, gevallen nuttig kunnen zijn.

| □ | □ | □ | □ | □ |
| --- | --- | --- | --- | --- |
| Volledig mee eens | Grotendeels mee eens | Weet niet | Grotendeels niet mee eens | Volstrekt niet mee eens |

12. Omdat ik deze formulieren maximaal wil benutten, wil ik ze liefst bij al mijn behandelingen gebruiken.

| □ | □ | □ | □ | □ |
| --- | --- | --- | --- | --- |
| Volledig mee eens | Grotendeels mee eens | Weet niet | Grotendeels niet mee eens | Volstrekt niet mee eens |

13. Ik denk dat mijn cliënten de relatie met mij als moeilijker ervaren als ik deze formulieren introduceer.

| □ | □ | □ | □ | □ |
| --- | --- | --- | --- | --- |
| Volledig mee eens | Grotendeels mee eens | Weet niet | Grotendeels niet mee eens | Volstrekt niet mee eens |

14. Het gebruik van deze formulieren zal vooral meer controle en beperking van therapeuten tot gevolg hebben en heeft weinig met goede therapie te maken.

| □ | □ | □ | □ | □ |
| --- | --- | --- | --- | --- |
| Volledig mee eens | Grotendeels mee eens | Weet niet | Grotendeels niet mee eens | Volstrekt niet mee eens |

15. De denkwijze van de cliënt heeft hem juist in de problemen gebracht. Het is daarom niet in het belang van de cliënt diens feedback -via de formulieren- een belangrijke rol te laten spelen in de behandeling.

| □ | □ | □ | □ | □ |
| --- | --- | --- | --- | --- |
| Volledig mee eens | Grotendeels mee eens | Weet niet | Grotendeels niet mee eens | Volstrekt niet mee eens |

16. Als behandelaar vind ik dat mijn eigen indruk van de sessie met de cliënten en de veranderingen die optreden, als leidraad van groter belang zijn dan de feedback die cliënten door middel van deze formulieren geven.

| □ | □ | □ | □ | □ |
| --- | --- | --- | --- | --- |
| Volledig mee eens | Grotendeels mee eens | Weet niet | Grotendeels niet mee eens | Volstrekt niet mee eens |

17. Als behandelaar vind ik het soms moeilijk om te weten of de cliënt voldoende baat heeft bij de behandelingen.

| □ | □ | □ | □ | □ |
| --- | --- | --- | --- | --- |
| Volledig mee eens | Grotendeels mee eens | Weet niet | Grotendeels niet mee eens | Volstrekt niet mee eens |

18. Met behulp van deze formulieren krijg ik een beeld van de therapeutische relatie

| □ | □ | □ | □ | □ |
| --- | --- | --- | --- | --- |
| Volledig mee eens | Grotendeels mee eens | Weet niet | Grotendeels niet mee eens | Volstrekt niet mee eens |

19.Met behulp van deze formulieren krijg ik een beeld van de veranderingen bij de cliënt.

| □ | □ | □ | □ | □ |
| --- | --- | --- | --- | --- |
| Volledig mee eens | Grotendeels mee eens | Weet niet | Grotendeels niet mee eens | Volstrekt niet mee eens |

**Dank je!**
