# Supplementary material for: Feedback-informed treatment in emergency psychiatry; a randomised controlled trial
Source: BMC Psychiatry. 2016 Apr 19;16:110. doi: 10.1186/s12888-016-0811-z (PMC4837581; doi:10.1186/s12888-016-0811-z)
Supplement: Additional file 2: — Adherence Survey. Adherence of therapists to feedback method (DOCX 14 kb) [file 12888_2016_811_MOESM2_ESM.docx]

Adherence Survey PCOMS (‘Beterwetermeter’)

F.J. van Oenen, 2010

Hoe vaak bespreek je de BWM?

(Kruis aan: bij hoeveel procent van de patiënten die binnenkomen met de Beterwetermeterformulieren, bespreek je deze daadwerkelijk in het gesprek?)

| 10 % | 20% | 30% | 40% | 50% | 60% | 70% | 80% | 90% | 100% |
| --- | --- | --- | --- | --- | --- | --- | --- | --- | --- |
|  |  |  |  |  |  |  |  |  |  |

Hoeveel aandacht besteed je aan het bespreken van de BWM?

(Kruis aan: als je de BWM gebruikt, hoeveel aandacht besteed je daar naar jouw idee aan tijdens het gesprek?)

| Amper | Weinig | Matig | Veel | Zeer Veel |
| --- | --- | --- | --- | --- |
|  |  |  |  |  |

Hoeveel minuten besteed je gemiddeld aan het bespreken van de BWM?

(Kruis aan: als je de BWM gebruikt, hoeveel minuten besteed je daar gemiddeld aan tijdens het gesprek?)

Outcome Rating Scale (‘Hoe gaat het met u?’ formulier)

| 1 | 2 | 3 | 4 | 5 | 6 | 7 | 8 | 9 | 10 |
| --- | --- | --- | --- | --- | --- | --- | --- | --- | --- |
|  |  |  |  |  |  |  |  |  |  |

Session Rating Scale (‘Hoe vond u de bijeenkomst?’ formulier)

| 1 | 2 | 3 | 4 | 5 | 6 | 7 | 8 | 9 | 10 |
| --- | --- | --- | --- | --- | --- | --- | --- | --- | --- |
|  |  |  |  |  |  |  |  |  |  |
